# Novel Artificial 5′UTR Increase Modified mRNA Translation When Injected into Mouse Heart

**DOI:** 10.3390/pharmaceutics17040490

**Published:** 2025-04-08

**Authors:** Ann Anu Kurian, Matteo Ghiringhelli, Eyal Shalom, Gayatri Mainkar, Magdalena M. Żak, Matthew Adjmi, Jeffrey Downey, Seonghun Yoon, Nicole Dubois, Filip K. Swirski, Lior Zangi

**Affiliations:** 1 Cardiovascular Research Institute, Icahn School of Medicine at Mount Sinai, New York, NY 10029, USA; ann.kurian@mssm.edu (A.A.K.); ghiringhelli.matteo@mssm.edu (M.G.); shalomey@gmail.com (E.S.); gaya.mainkar@icahn.mssm.edu (G.M.);magdalena.zak@mssm.edu (M.M.Ż.); matthew@adjmi.com (M.A.); jeffrey.downey@mssm.edu (J.D.); seonghun.yoon@mssm.edu (S.Y.); filip.swirski@mssm.edu (F.K.S.); 2 Department of Genetics and Genomic Sciences, Icahn School of Medicine at Mount Sinai, New York, NY 10029, USA; 3 Black Family Stem Cell Institute, Icahn School of Medicine at Mount Sinai, New York, NY 10029, USA; 4 Cell, Development/Regenerative Biology, Icahn School of Medicine at Mount Sinai, New York, NY 10029, USA; nicole.dubois@mssm.edu; 5 Marc and Jennifer Lipschultz Precision Immunology Institute, Icahn School of Medicine at Mount Sinai, New York, NY 10029, USA

**Keywords:** modified RNA (modRNA), 5′ untranslated region, cardiac gene therapy, translation efficiency, synthetic RNA motif

## Abstract

**Background/Objectives:** Modified messenger RNA (modRNA) is a promising gene delivery method used to upregulate genes in cardiac tissue, with applications in both clinical and preclinical settings to prevent cardiac remodeling after ischemic injury. The 5′ untranslated region (5′UTR) plays a crucial role in regulating the translation efficiency of mRNA into functional proteins. Due to the high production cost and short half-life of modRNA, it is essential to identify novel 5′UTR designs that enhance modRNA translation in the heart. **Methods:** Here, we present an artificial 5′UTR, termed “Top Heart 5′UTR”, designed based on ribonucleotide frequency analyses of 1000 genes highly expressed in the heart. This novel artificial 5′UTR contains a unique 20-nucleotide sequence, consisting of 11 previously uncharacterized nucleotides (CCCCCGCCCCC) and 9 well-described nucleotides from the Kozak sequence upstream of the start codon (ATG). **Results:** This design significantly improves modRNA translation efficiency in cardiomyocytes (CMs) and heart cells both in vitro and in vivo. Specifically, the Top Heart 5′UTR increases translation efficiency by approximately 30–60% in both mouse and human CMs compared to a standard 5′UTR control. Moreover, the artificial 5′UTR induces a 2–2.5 times higher translation of modRNA in the mouse heart 24 and 48 h post-delivery. **Conclusions:** Our findings may contribute to the development of a superior modRNA platform for use in preclinical and clinical studies, potentially allowing reduced dosages or increased gene expression at the same dosage level. This approach can be extended to identify optimized 5′UTRs for various cell types or organs, including applications in cancer therapies.

## 1. Introduction

Messenger RNA (mRNA) is a single-stranded RNA molecule that corresponds to the genetic sequence of a gene, serving as a template for protein production. A mature eukaryotic mRNA molecule comprises several key components: a 5′ cap structure, a 5′ untranslated region (UTR), an open reading frame (ORF), a 3′UTR, and a poly(A) tail at the 3′ end [1]. Each of these elements plays a distinct role in regulating the mRNA’s function. The 5′UTR, in particular, is crucial for controlling gene expression by influencing mRNA stability, localization, and translation efficiency [2].

The translation of eukaryotic mRNA begins with the recruitment of the ribosome to the 5′ cap. This process is initiated when the 7-methylguanosine (m7G) cap at the 5′ end interacts with eukaryotic initiation factors (eIFs), leading to the assembly of the translation initiation complex [3]. Canonical cap-mediated translation involves the recruitment of the 40S ribosomal subunit to the m7G cap via eIF4E, a key component of the trimeric eIF4F complex. eIF4G and eIF4A also interact with eIF4E, as well as the poly(A)-binding protein (PABP) at the 3′UTR, creating a circular mRNA structure that facilitates efficient translation. The eIF3 and eIF4G factors recruit the 43S pre-initiation complex, which binds near the cap and scans along the 5′UTR in the 5′ to 3′ direction until it encounters the start codon (AUG), where translation begins [3].

The length and sequence of the 5′UTR significantly influence translation efficiency. In humans, the average size of a 5′UTR is approximately 200 nucleotides, but shorter UTRs (around 40 nucleotides) are advantageous for genes with high expression demands, such as ribosomal protein genes, as they enhance translation efficiency [2,4].

In recent years, advancements in RNA technology have led to the development of modified mRNA (modRNA), which has emerged as a promising tool for improving gene expression in therapeutic contexts. modRNA, developed by Dr. Katalin Karikó and Dr. Drew Weissman in 2005, represents a significant step forward by circumventing the innate immune response. This was achieved by substituting uridine with pseudouridine, thereby preventing recognition by innate immune receptors, such as TLR7/8 [5,6]. These modifications make modRNA more stable and capable of enhancing gene expression without the cytotoxicity associated with unmodified mRNA. Their groundbreaking discovery eventually earned them the 2023 Nobel Prize in Medicine. modRNA has since been utilized in various clinical and preclinical settings, notably in the development of COVID-19 vaccines, significantly expanding its applications beyond vaccines [7,8]. Our group, along with others, has explored modRNA-based cardiac therapies to promote cardiac repair following ischemic injury by transiently upregulating specific genes of interest in the heart [9,10,11,12,13]. Cardiac applications of modRNA typically involve using an artificial 5′UTR to improve translation efficiency. The 5′UTR plays a critical role in determining how effectively the modRNA can be translated within cardiac cells. A commonly used artificial 5′UTR is 44 nucleotides in length, which has been shown to enhance modRNA performance in preclinical studies by promoting efficient translation within cardiomyocytes and improving therapeutic outcomes [14]. However, there remains a need to further optimize these 5′UTRs specifically for cardiac tissues to maximize therapeutic efficacy. This is particularly important given the context of ischemic heart disease (IHD), which is the leading cause of death globally for both men and women [15]. Effective treatment options that support cardiac regeneration and limit damage post-injury are essential for improving patient outcomes. The transient nature of gene expression achieved with modRNA administration makes it an ideal candidate for treating myocardial infarction (MI) and IHD. modRNA can provide temporary, high-level expression of therapeutic proteins that promote cardiomyocyte proliferation, reduce post-injury remodeling, and enhance cardiovascular regeneration by increasing capillary density after a heart attack [16]. Despite its potential, the short expression period and high production cost of modRNA necessitate the identification of optimized 5′UTRs to enhance translation efficiency and therapeutic effects in cardiac tissue. In a previous study, we used transcriptomic and proteomic analyses of heart tissues to identify potential 5′UTRs and their functional elements that could enhance modRNA translation after ischemic injury [17]. We demonstrated that the 5′UTR from the fatty acid metabolism gene carboxylesterase 1D (Ces1d) improved luciferase (Luc) modRNA translation by two-fold in the heart post-MI. Further analysis revealed a specific RNA element within the Ces1d 5′UTR, termed element D (ucagagacccacagagccc), which improved modRNA translation by 2.5-fold compared to a modRNA with a standard artificial 5′UTR.

Building on these findings, the current study aims to identify 5′UTRs for modRNA that enhance translation specifically in cardiac tissue. This was accomplished by analyzing the nucleotide frequency of adenine (A), uracil (U), cytosine (C), and guanine (G) at each of the 40 positions upstream of the start codon in the ORFs of genes expressed in the heart. Our goal is to pinpoint specific 5′UTR sequences that can optimize modRNA translation and improve therapeutic outcomes for cardiac applications.

## 2. Materials and Methods

### 2.1. Identification of 5′UTR Sequences: Top Heart, Top CMs, and Top Elevated Heart

We analyzed RNA expression data from the Human Protein Atlas to identify potential 5′UTRs from three groups of genes: the top 1000 genes expressed in the heart (“Top Heart”), the top 1000 genes expressed in cardiomyocytes (“Top CMs”), and the top 348 genes elevated post-heart attack (“Top Elevated Heart”). For each gene, the most representative mRNA sequence was selected, and the open reading frame (ORF) data were obtained from NCBI. Genes with 3′UTRs shorter than 40 base pairs were filtered out, according to findings previously published suggesting that a 5′UTR length of approximately 40 nucleotides, with minimal secondary structure, balances the need to avoid leaky scanning associated with very short UTRs and the potential formation of inhibitory secondary structures in longer UTRs [18,19]. The top 1000 genes by normalized transcripts per million (nTPM) were selected from each group, and the 40 nucleotides upstream of the start codon were extracted. A probability matrix was built based on nucleotide frequencies at each position within the 5′UTR sequences, and a consensus sequence was generated by selecting the most frequent nucleotide at each position. This analysis aimed to identify shared nucleotide patterns and regulatory motifs in the 5′UTRs of these genes, revealing insights into post-transcriptional regulation in the heart, both under normal conditions and following myocardial infarction (Table 1).

### 2.2. Mathematical Method for Consensus Sequence Calculation

To identify the consensus sequence of the most frequently expressed genes in the heart, the following steps were applied.

1. Sequence set definition: Let S = {s1, s2, …, sN} represent the set of RNA sequences, where *N* is the total number of sequences analyzed. Each sequence sN is defined as sN = (*b*1, *b*2, …, *bM*), where *b_j_* ∈ (*18T*) represents the nucleotide at position *j*, and *L* is the sequence length (e.g., 40 base pairs).
2. Frequency matrix calculation: For each position *i* ∈ {1, 2, …, *M*}, the frequency *f_i_*(*b*) of each nucleotide *b* ∈ {*A*, *C*, *G*, *T*} was computed as
  $$f_{ij}(b)\;\frac{Count\;of\;nucleotide\;bj\;at\;position\;j}{N}$$
  where Count of nucleotide *b* at position *j* is the total number of sequences containing nucleotide *b* at position *j*, and *N* is the total number of sequences. The resulting frequency matrix is normalized such that
  $$\sum_{b\;\in \;\left\{A,C,G,T\right\}}f_{ij}\left(b\right)=1\;\forall i\;\in \;\left\{1,2,\ldots ,L\right\}$$

1. Consensus sequence derivation: The consensus nucleotide at each position was determined by selecting the nucleotide with the highest frequency:
  $$b_{j}*=\begin{array}{c} \arg\;\max{\;f}_{i\;}\left(b\right)\\ b\;\in \;\left\{A,C,G,T\right\} \end{array}$$
  where *arg max* identifies the nucleotide *b_j_* that maximizes *f_i_*(*b*). If two or more nucleotides had the same maximum frequency, one of them was chosen arbitrarily.
2. Handling missing or ambiguous data: If no nucleotide was observed at a given position *j* (i.e., *f_i_*(*b*) *=* 0 for all ∀ *b* ∈{*A*, *C*, *G*, *T*}), the position was marked with an ambiguous nucleotide symbol N.
3. Final consensus sequence: The consensus sequence *C* = (*b*_1_*, *b*_2_*,…, *b*_L_*) was constructed by concatenating the consensus nucleotide b_j_* for all positions *i*.

### 2.3. Construction of DNA Templates and Synthesis of Synthetic mRNA

Clean PCR products generated from plasmid templates of nGFP or luciferase (Luc), each carrying different 5′UTRs, were purchased from GenScript and used as templates for mRNA synthesis. modRNAs were synthesized via in vitro transcription using a customized ribonucleoside blend consisting of Cleancap AG (Trilink Biotechnologies, San Diego, CA, USA), GTP, ATP, CTP (Life Technologies, Carldbad, CA, USA), and N1-methylpseudouridine-5′-triphosphate (Trilink Biotechnologies, San Diego, CA, USA). The synthesized mRNA was purified either using the MEGAclear kit (Life Technologies, Carldbad, CA, USA) according to the manufacturer’s instructions or with Amicon Ultra-4 Centrifugal Filter Units (Millipore Sigma (St. Louis, MO, USA). mRNA quantification was performed using a NanoDrop spectrometer (Thermo Scientific, Waltham, MA, USA).

### 2.4. Neonatal Mouse Cardiomyocyte Isolation

All animal procedures were performed according to protocols approved by the Icahn School of Medicine at Mount Sinai Institutional Care and Use Committee (ref. number IACUC-2015-0043). Cardiomyocytes were taken from C57BL/6 2-day-old pups using the Pierce Cardiomyocyte Isolation Kit (Thermo Scientific, Waltham, MA, USA). Cardiomyocytes were seeded at a density of 5 × 10^5^ cells per well in a 24-well plate, following the kit’s recommended protocol.

### 2.5. Differentiation of Human Induced Pluripotent Stem Cells (hiPSCs)

Human induced pluripotent stem cells (hiPSCs, H9) were differentiated along a cardiac lineage. hiPSCs were maintained in E8 medium and passaged every 4–5 days onto Matrigel-coated plates. To generate embryoid bodies (EBs), hiPSCs were treated with 1 mg/mL collagenase B (Roche, Basel, Switzerland) for 30 min or until the cells detached. The detached cells were collected, centrifuged at 1300 rpm for 3 min, and resuspended in differentiation medium containing RPMI (Gibco, Waltham, MA, USA), 2 mmol/L L-glutamine (Invitrogen, Wlatham, MA, USA), 4 × 10^4^ monothioglycerol (MTG, Sigma, Kawasaki, Japan), 50 μg/mL ascorbic acid (Sigma, Kawasaki, Japan), and 150 μg/mL transferrin (Roche, Basel, Switzerland). Differentiation medium was supplemented with 2 ng/mL BMP4 and 3 μmol/L Thiazovivin (Millipore Sigma, St. Louis, MO, USA) on day 0. EBs were maintained in six-well ultra-low attachment plates (Corning, Corning, NY, USA) at 37 °C in 5% CO_2_, 5% O_2_, and 90% N_2_. On day 1, the medium was changed to differentiation medium supplemented with 20 ng/mL BMP4 and 20 ng/mL Activin A (R&D Systems, Minneapolis, MN, USA). On day 4, the medium was replaced with differentiation medium containing 5 ng/mL VEGF (R&D Systems, Minneapolis, MN, USA) and 5 μmol/L XAV939 (Stemgent, Beltsville, MD, USA). From day 8 onward, media changes were performed every 5 days using differentiation medium without additional supplements.

### 2.6. FACS Analysis for nGFP Expression

Isolated mouse or human cardiomyocytes were incubated for 48 h in DMEM medium containing 5% horse serum plus cytosine arabinoside (Ara-C). After incubation, cells were transfected with nGFP modRNAs carrying different 5′UTRs. After 24 h, cells were collected, and fluorescence-activated cell sorting (FACS) analysis was performed to evaluate GFP expression across the different treatment groups. Shortly, transfected murine neonatal cardiomyocytes or organoid-derived human cardiomyocytes were incubated at 37 °C 5% CO_2_ for 5 min in 1mL of 0.25% Trypsin-EDTA. After 5 min, the trypsin was pipetted up and down gently to fully dislodge the cells. Cardiomyocytes were then put into FACS tubes, washed with FACS buffer (0.5% BSA and 2 mM EDTA in PBS), and resuspended in 300 µL of FACS buffer for acquisition. Cells were acquired on a Cytek Aurora (Cytek, Fremont, CA, USA) and analyzed with FlowJo software Version 10. Non-transfected cardiomyocytes served as staining controls and the basis for GFP MFI analyses.

### 2.7. Bioluminescence Imaging In Vivo

All animal procedures were performed according to protocols approved by the Icahn School of Medicine at Mount Sinai Institutional Care and Use Committee (ref. number IACUC-2015-0043). Here, 30 μg samples of Luciferase (Luc) modRNA constructs, each with different 5′UTRs, were directly injected into the myocardium of CFW 12-week-old mice. The bioluminescence imaging of the injected mice was performed at 24 and 48 h post-modRNA delivery. Prior to imaging, mice were anesthetized with isoflurane (Abbott Laboratories, Lake County, IL, USA), and D-Luciferin Potassium Salt (PerkinElmer, Waltham, MA, USA) was administered intraperitoneally at 150 mg/kg body weight. The mice were then imaged using an IVIS100 (Revvit, Waltham, MA, USA) charge-coupled device (CCD) imaging system every 2 min until the luciferase signal reached a plateau. Imaging data were analyzed and quantified using Living Image software version 2.0. Cardiac tissues from mice injected with only luciferin were used as baseline controls for background luciferase expression.

### 2.8. Comparison of Free Energy Secondary Structures

The results have been computed using RNAfold 2.6.3. An equivalent command line call would have been RNAfold-p-d2--noLP < sequence1.fa > sequence1.out.

### 2.9. Statistical Analysis

Statistical significance was determined by One-way ANOVA and Tukey’s Multiple Comparison Test. A *p*-Value < 0.05 was considered significant. All graphs represent average values, and values were reported as mean ± standard error of the mean. ****, *p* < 0.0001, ***, *p* < 0.001, **, *p* < 0.01, *, *p* < 0.05; ns, not significant.

## 3. Results

### 3.1. Evaluation of 5′UTR Translation Efficiency in Neonatal Mouse Cardiomyocytes

**Figure 1:**
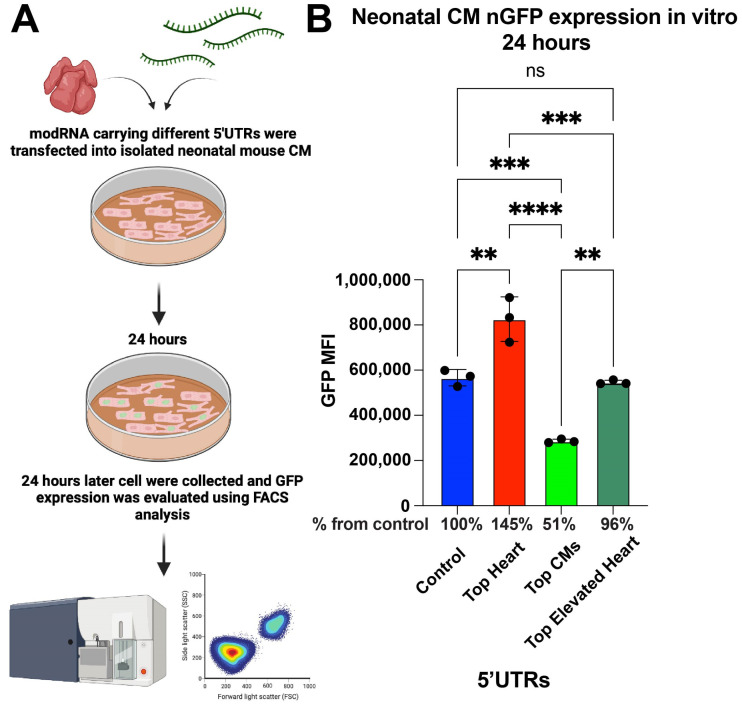
nGFP modRNA carrying 5′UTR Top Heart shows significant higher translation post-delivery into isolated neonatal CM over other 5′UTRs. (**A**) Neonatal mouse CMs were isolated from P1 mice and isolated cells were plated in a 6-well plate. At day four after isolation, cells were transfected with nGFP modRNA carrying different 5′UTRs. One day later, they were collected, and FACS analysis was used to evaluate GFP expression. (**B**) The quantification of GFP Median Fluorescence Intensity (MFI) was based on the experiment on A (n = 3). One-way ANOVA and Tukey’s Multiple Comparison Test were used. ****, *p* < 0.0001, ***, *p* < 0.001, **, *p* < 0.01; ns, not significant.

To assess the abilities of different 5′UTRs to promote modRNA translation, neonatal cardiomyocytes (CMs) were isolated from postnatal day 1–3 mice and transfected with nGFP modRNA constructs containing various 5′UTRs. After 24 h, fluorescence-activated cell sorting (FACS) analysis was performed to compare GFP expression levels (Figure 1A). The results show that nGFP modRNA carrying the Top Heart 5′UTR exhibited 45% higher expression compared to the control 5′UTR. Interestingly, the Top CMs 5′UTR led to a significantly lower expression, achieving only 51% of the expression observed in the control, indicating a reduced translation efficiency in neonatal CMs (Figure 1B).

### 3.2. In Vivo Evaluation of modRNA Translation Efficiency

**Figure 2:**
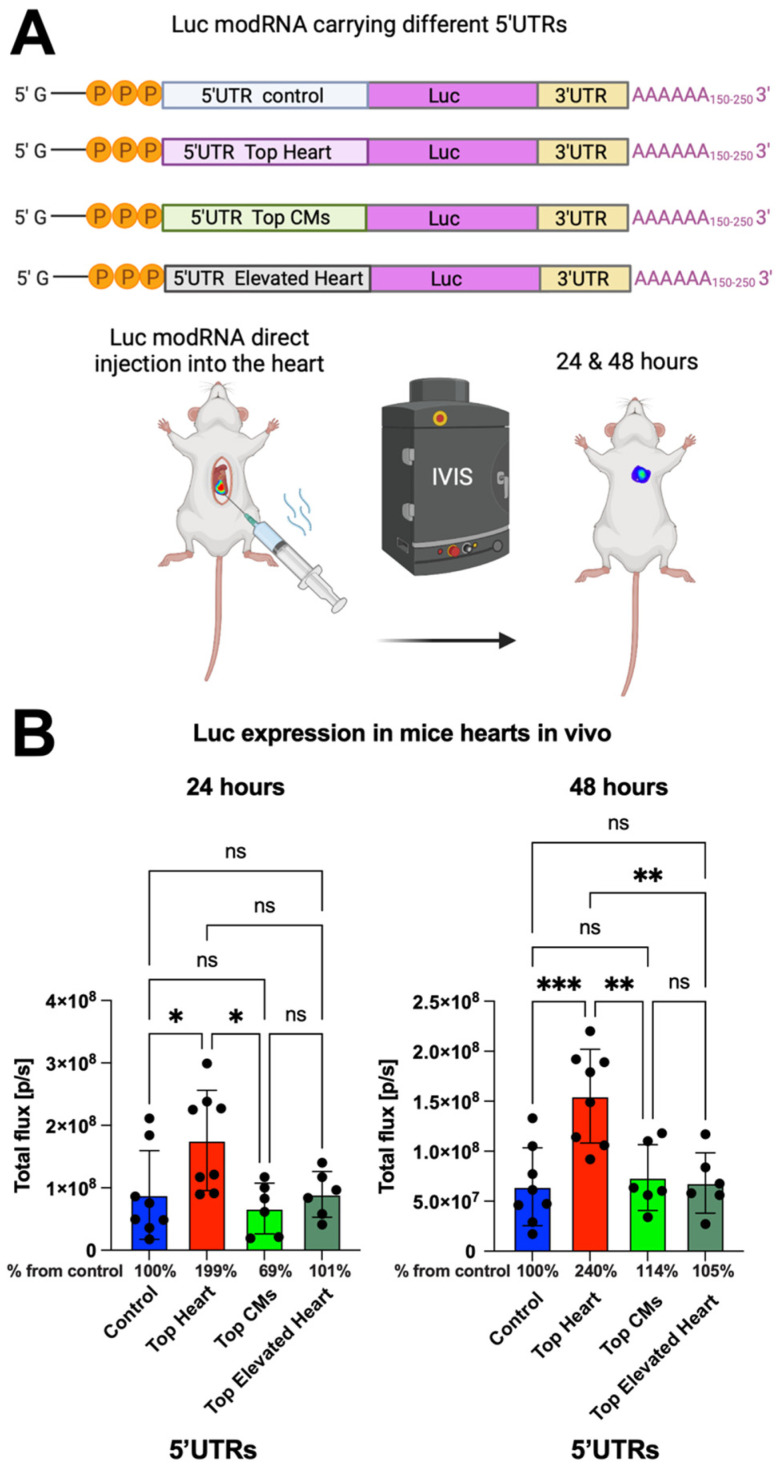
Luc modRNA carrying 5′UTR Top Heart has significant higher translation post-delivery directly into mouse heart over other 5′UTRs. (**A**) Delivery of naked Luc modRNA carrying different 5′UTRs directly into the heart in an open chest surgery. The IVIS system was used to evaluate Luc expression in the heart one and two days post-delivery. (**B**) The quantification of Luc expression based on the experiment on (**A**) (n = 6–8). One-way ANOVA and Tukey’s Multiple Comparison Test were used. ***, *p* < 0.001, **, *p* < 0.01, *, *p* < 0.05; ns, not significant.

To further evaluate the translation efficiency of the different 5′UTRs in vivo, we conducted open-chest surgery on CFW mice and directly injected luciferase (Luc) modRNA constructs containing the different 5′UTRs into the myocardium. Bioluminescence imaging was performed at 24 and 48 h post-injection to measure Luc expression (Figure 2A). We observed that modRNA containing the Top Heart 5′UTR produced 2-fold higher gene expression after 24 h and approximately 2.4-fold higher expression after 48 h compared to the control 5′UTR (Figure 2A,B). This suggests that the Top Heart 5′UTR significantly enhances modRNA translation efficiency for cardiac applications.

### 3.3. Analysis of Sequence Differences and Functional Implications

Although the sequences of the Top Heart and Top CMs 5′UTRs were similar, the Top CMs 5′UTR consistently showed lower translation efficiency. We identified two specific regions—Area A (first 20 nucleotides) and Area B (last 20 nucleotides)—within the Top Heart 5′UTR that differed from the Top CMs 5′UTR (Table 2). To further evaluate the significance of these regions, we generated new modRNA constructs, each containing different combinations of these areas: Top Heart A 50% 5′UTR and Top Heart B 50% 5′UTR. We synthesized two new modRNA constructs; one containing the first 20 nucleotides from the Top Heart 5′UTR and the last 20 nucleotides from the Top CMs 5′UTR (“Top Heart A 50% 5′UTR”), and the other containing the last 20 nucleotides from the Top Heart 5′UTR and the first 20 nucleotides from the Top CMs 5′UTR (“Top Heart B 50% 5′UTR”).

**Figure 3:**
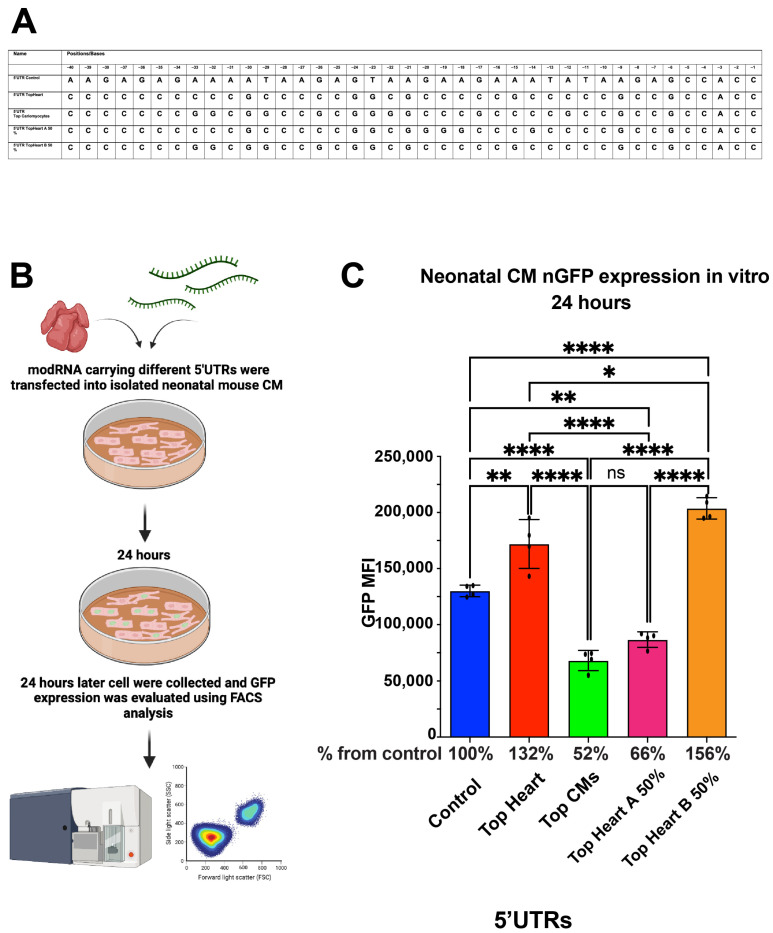
nGFP modRNA carrying 5′UTR Top Heart or carrying the last 20 nucleotides of 5′UTR Top Heart has a significantly higher translation post-delivery into isolated neonatal CMs over other 5′UTRs. (**A**) 5′UTRs of different nGFP modRNA were compared for their ability to promote modRNA translation into neonatal CMs. (**B**) Neonatal mouse CMs were isolated from P1 mice and isolated cells were plated in a 24-well plate. At day four post isolation, cells were transfected with nGFP modRNA carrying different 5′UTRs (see all 5′UTRs used in (**A**)). One day later, cells were collected, and FACS analysis was used to evaluate GFP expression. (**C**) Quantification of GFP Median Fluorescence Intensity (MFI) based on the experiment on B using the 5′UTR that presented in (**A**) (n = 4). One-way ANOVA and Tukey’s Multiple Comparison Test were used. ****, *p* < 0.0001, **, *p* < 0.01, *, *p* < 0.05; ns, not significant.

We found that nGFP modRNA constructs containing either the full Top Heart 5′UTR or the last 20 nucleotides of the Top Heart 5′UTR (Top Heart B 50%) exhibited significantly higher translation efficiency compared to the control 5′UTR or the Top CMs 5′UTR in neonatal mouse CMs (Figure 3). These data suggest that Area B is sufficient and necessary to enhance modRNA translation efficiency in neonatal mouse CMs.

### 3.4. Evaluation in Human iPSC-Derived Cardiomyocytes

**Figure 4:**
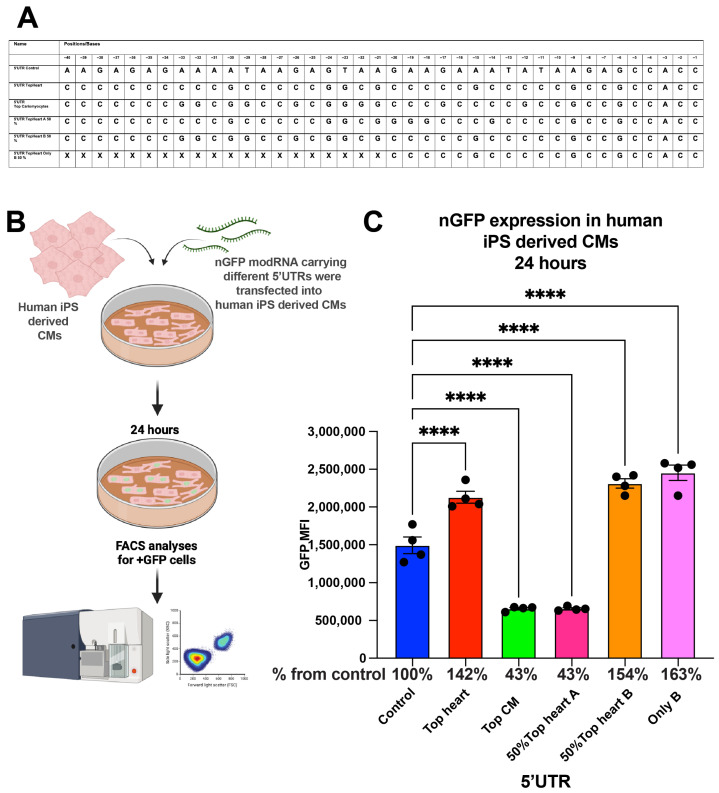
nGFP modRNA carrying 5′UTR and the last 20 nucleotides of 5′UTR Top Heart has significantly higher translation post-delivery into human iPS-derived CMs over control 5′UTR. (**A**) 5′UTRs of different nGFP modRNA were compared for their ability to promote modRNA translation into Human iPS-derived CMs. (**B**) Human iPS-derived CMs were plated in 24 well plate. At day four post isolation, cells were transfected with nGFP modRNA carrying different 5′UTRs (see all 5′UTRs used in (**A**)). One day later, cells were collected, and FACS analysis was used to evaluate GFP expression. (**C**) Quantification of GFP Median Fluorescence Intensity (MFI) based on the experiment on B using the 5′UTR that was presented in (**A**) (n = 4). One-way ANOVA and Tukey’s Multiple Comparison Test were used. ****, *p* < 0.0001.

To explore the applicability of our findings to human cardiomyocytes, we compared the translation efficiency of nGFP modRNA constructs with different 5′UTRs in human induced pluripotent stem cell (iPSC)-derived cardiomyocytes (Figure 4). Similar to our findings in neonatal mouse CMs, the Top CMs 5′UTR, Top Heart B 50% 5′UTR, and the “Only B” 5′UTR increased nGFP expression by 42% and 63%, respectively, compared to the control 5′UTR at 24 h post-transfection.

Overall, our results suggest that the Top Heart 5′UTR and the Top Heart B 50% 5′UTR significantly enhance modRNA translation efficiency in both mouse and human cardiomyocytes compared to the control 5′UTR. Notably, Area B within the Top Heart 5′UTR appears to play a crucial role in this enhancement. These findings have significant implications for the design of efficient modRNA constructs for cardiac applications, both in vitro and in vivo.

## 4. Discussion

The 5′ untranslated region (5′UTR) of mRNA is a crucial element for efficient mRNA translation initiation. The amount of modRNA used for the COVID-19 vaccine in humans ranged from 30–100 µg per patient [8]; however, in ducing cardiovascular regeneration in human clinical trials post-ischemic injury required approximately 3000 µg of modRNA per patient [20]. Optimizing the cardiac 5′UTR to enhance modRNA translation could significantly reduce the dosage needed per patient, thereby decreasing costs and minimizing potential side effects.

**Figure 5:**
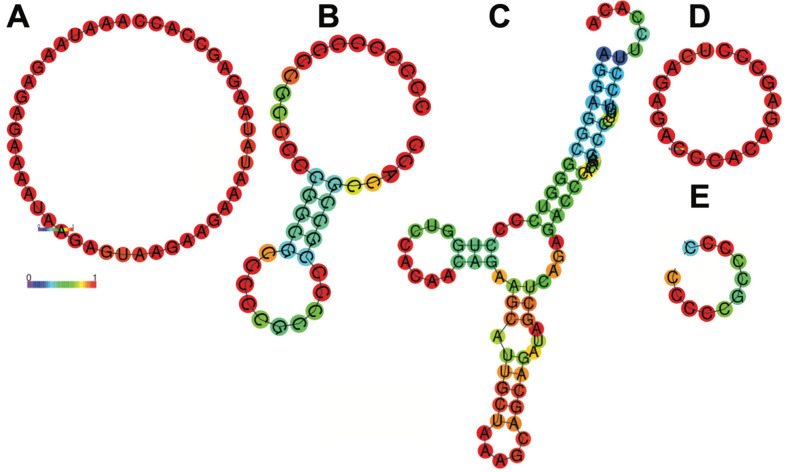
Secondary structure of control 5′UTR (**A**), Top Heart (**B**), Cesd1D (**C**), Element D (**D**) and only B (**E**) 5′UTRs. In this schematic, the colors of the base pair indicate the likelihood of a base pair forming in the RNA secondary structure. Red shows high probability, close to 1 (strongly predicted to pair). Yellow/orange show intermediate probabilities. Blue shows low probability, close to 0 (unlikely to pair). In the ViennaRNA package, a probability of 1 means that a specific base pair always forms in the ensemble of all possible RNA secondary structures, weighted by their thermodynamic stability (Boltzmann distribution). Results have been computed using RNAfold 2.6.3. An equivalent command line call would have been RNAfold-p-d2--noLP < sequence1.fa > sequence1.out.

In our study, we successfully created an artificial 5′UTR based on the frequencies of four ribonucleotides at different positions upstream of the start codon, using sequences from 1000 highly expressed human cardiac genes. This artificial 5′UTR significantly improved modRNA translation efficiency in both mouse and human cardiomyocytes, as well as in the mouse heart (Figure 1, Figure 2, Figure 3 and Figure 4 and Table 1, Table 3 and Table 4). Our artificial cardiac 5′UTR demonstrated superior performance compared to the control 5′UTR previously used in cardiac preclinical and clinical studies. Previous investigations have attempted to optimize 5′UTRs for mRNA therapeutics by utilizing randomized 5′UTR libraries and evaluating gene expression in various cell lines [19,20,21,22]. Others have suggested that shorter 5′UTRs may lead to enhanced expression efficiency [23,24]. A recent study [25] employed language models to analyze endogenous 5′UTRs from multiple species, combining supervised information such as secondary structure and minimum free energy to predict translation efficiency. This approach successfully identified 211 novel 5′UTRs with high predicted translation efficiency, but our approach offers a simpler alternative. By focusing on the conservation of beneficial RNA motifs, such as those seen in the poly(A) tail or Kozak sequence, we were able to create a sequence optimized for cardiac gene expression. We identified an artificial 20-nucleotide RNA motif, termed “Only B 5′UTR,” consisting of 11 unique nucleotides (cccccgccccc) and 9 nucleotides from the Kozak sequence (gccgccacc) upstream of the start codon. This motif was sufficient and necessary to increase modRNA translation by 30–60% in mouse and human cardiomyocytes and by 2–2.5-fold in the mouse heart. Our previous approach involved using transcriptomic and proteomic data from mouse hearts post-MI to identify potential 5′UTRs that could enhance translation in ischemic cardiac tissue. This resulted in the discovery of a short 19-nucleotide sequence (termed “Element D”) that provided a 2-fold increase in modRNA translation [17]. Other studies have utilized in silico algorithms to match cell-specific protein expression with predictive models. While these approaches yielded comparable results in terms of translation efficiency, they were primarily conducted in non-cardiomyocyte eukaryotic cells [22,26,27]. Interestingly, novel artificial 5′UTR and Element D both demonstrated significant enhancements of modRNA translation compared to the control 5′UTR. However, the sequences differed substantially—“Only B” was composed almost entirely of cytosine (90%), whereas Element D contained only 42% cytosine. The comparison of free energy secondary structures revealed that the control 5′UTR had the most favorable folding energy, which might indicate that secondary structure alone does not dictate translation efficiency (Figure 5). Instead, specific motifs, such as those identified in this study, may play a larger role in promoting efficient translation.

Unlike previous studies that used immortalized cell lines for evaluation [19,21,22,23,24,25], we tested our novel 5′UTRs in primary neonatal mouse cardiomyocytes and iPSC-derived human cardiomyocytes (Figure 1 and Figure 3). Our data show that the Top Heart 5′UTR and the Top Heart B 50% 5′UTR were superior in promoting translation compared to the control. This finding is promising for their potential use in human cardiac settings. Surprisingly, modRNA containing the Top CMs and Top Elevated Heart 5′UTRs resulted in significantly lower gene expression compared to modRNA containing the Top Heart 5′UTR. This difference allowed us to identify the “Only B” sequence as the primary driver of translation efficiency as an artificial sequence. Although “Only B” does not share significant primary sequence homology with the native 5′UTRs of TNNT2 in either human or mouse, its enhanced performance likely results from optimized sequence features—such as GC richness, minimal secondary structure, and favorable ribosome accessibility—rather than evolutionary conservation. This suggests that synthetic UTRs can be designed to surpass endogenous elements in driving efficient translation across species. We believe that cells in different organs have evolved to preferentially use specific RNA motifs (such as “Only B”) that improve the translation process, making the proposed approach broadly applicable for different tissues. Our study identifies the “Only B” 5′UTR as an effective RNA motif for enhancing modRNA translation in cardiomyocytes and in the mouse heart. It remains unclear whether this RNA motif will show the same translation enhancement in an ischemic heart model compared to other 5′UTRs, such as Ces1d or Element D. Additionally, it will be important to evaluate whether this motif enhances translation in other tissues beyond the heart. Moreover, we acknowledge the limited value of the current in vitro models, which lack cellular diversity and therefore cannot fully replicate the phenotypic interactions that occur within a multicellular environment. To better address these limitations, future studies will explore more complex in vivo and ex vivo approaches, such as cardiac thin slices or perfused heart preparations using a Langendorff setup [28,29]. These models may offer greater insights into the mechanisms driving enhanced protein production in the context of cell-to-cell interactions. However, due to the limited number of studies combining these techniques with mRNA expression analysis, we chose to first investigate our constructs using well-established classical assays as an initial step in this process. Moving forward, we plan to examine the ability of the “Only B” 5′UTR to upregulate previously established therapeutic genes, such as Pyruvate Kinase M2 (PKM2) or Acid Ceramidase in iPSC-derived human cardiac cells, cardiac slices and ex vivo perfused hearts in normothermic conditions. The evaluation of the novel artificial 5′UTR’s efficacy in promoting modRNA translation will be crucial in human clinical trials, as well as large animal models, under both healthy and ischemic conditions. The clinical translation of modified mRNA (modRNA) therapies has gained significant momentum in recent years. One notable example is the EPICCURE trial [20], where direct intramyocardial injection of VEGF-A modRNA during coronary artery bypass grafting was assessed for its potential to promote angiogenesis and improve myocardial perfusion. This first-in-human trial demonstrated the safety and feasibility of therapeutic modRNA delivery in a cardiac setting, underscoring the importance of optimizing every element of the mRNA construct, especially untranslated regions (UTRs), to maximize translational output and therapeutic efficacy. In conclusion, we designed and evaluated a novel artificial 5′ untranslated region (5′UTR), termed “Only B”, to enhance the translation efficiency of modified mRNA (modRNA) in cardiac cells. Using a bioinformatic approach based on nucleotide frequency analysis from highly expressed cardiac genes, we generated optimized synthetic UTR sequences that were experimentally validated in both neonatal mouse cardiomyocytes and human iPSC-derived cardiomyocytes. Among these, the “Only B” 5′UTR demonstrated superior translation efficiency, leading to a 30–60% increase in protein production in vitro and up to 2.5-fold enhancement in vivo in the mouse heart. Importantly, although “Only B” does not share significant homology with native 5′UTRs from cardiac genes such as TNNT2, its high GC content, minimal secondary structure, and efficient ribosome recruitment likely contribute to its translational advantage. These findings emphasize the functional relevance of synthetic UTR design for therapeutic mRNA applications. Our results provide a strong foundation for the further development of tissue-specific modRNA therapeutics. Future studies will aim to assess the performance of “Only B” in ischemic conditions and larger animal models, as well as in delivering clinically relevant therapeutic genes. This approach may significantly reduce the required modRNA dosage, decrease manufacturing costs, and enhance safety for future human applications in cardiovascular gene therapy.

## Figures and Tables

**Table 1:** Consensus table for the identification of 5′UTR Top heart, Top CMs and Top Elevated Heart. The frequency of different mRNA ribonucleotides was assessed. We evaluate 40 ribonucleotides positions before the start codon. The most frequent ribonucleotide in the 1000 or 348 genes tested was selected for each position, where the ‘Manual Completion’ line shows the final sequence obtained, respectively, for Top Heart, Top CMs, and Top Elevated Heart.

Top 1000 genes sorted by total expression in heart (n = 1000)																																								
Base/Pos	−40	−39	−38	−37	−36	−35	−34	−33	−32	−31	−30	−29	−28	−27	−26	−25	−24	−23	−22	−21	−20	−19	−18	−17	−16	−15	−14	−13	−12	−11	−10	−9	−8	−7	−6	−5	−4	−3	−2	−1
A	16	16	14	14	17	15	16	16	16	15	16	18	15	15	14	15	14	17	15	17	18	17	16	17	20	17	19	19	17	21	24	21	16	19	17	12	21	52	28	15
T	19	21	20	20	21	22	21	21	19	18	17	19	18	19	18	17	18	19	17	19	17	18	17	17	18	18	15	18	17	16	17	14	16	15	15	19	7	3	10	5
G	29	29	31	29	28	28	27	30	31	31	35	31	31	32	34	32	34	32	30	34	30	29	33	27	26	33	31	29	30	25	24	39	28	25	45	27	18	41	16	25
C	36	34	35	37	35	34	36	32	34	35	32	32	35	34	35	35	33	32	38	30	34	36	34	38	37	32	35	34	35	38	35	25	40	41	23	42	54	5	47	54
tot	C	C	C	C	C	C	C	C	C	C	G	C	C	C	C	C	G	G	C	G	C	C	C	C	C	G	C	C	C	C	C	G	C	C	G	C	C	A	C	C
Top 1000 genes sorted by total expression in cardiomyocytes (n = 1000)																																								
A	19	17	17	17	16	16	16	16	16	16	17	18	15	18	16	18	17	19	17	18	21	18	16	19	18	17	19	19	19	22	22	21	18	21	19	17	23	51	31	17
T	20	20	21	20	22	23	23	21	20	20	20	21	21	20	20	20	19	20	16	19	18	18	18	19	18	19	16	19	19	17	17	18	16	18	14	17	9	3	11	6
G	30	30	30	30	28	28	27	33	33	32	32	31	32	31	34	29	34	32	32	34	31	32	33	28	28	31	33	28	31	25	27	38	29	26	48	27	21	41	16	26
C	32	32	31	33	35	33	34	30	31	32	32	30	32	32	30	34	30	29	35	29	30	31	32	35	35	33	32	33	31	35	34	23	37	36	19	39	46	5	42	50
tot	C	C	C	C	C	C	C	G	G	C	G	G	C	C	G	C	G	G	C	G	G	G	G	C	C	C	G	C	C	C	C	G	C	C	G	C	C	A	C	C
Elevated genes in heart (n = 348)																																								
A	16	18	18	18	20	18	18	21	19	19	22	16	17	17	20	18	19	18	18	18	22	19	15	22	20	20	15	14	18	23	19	22	21	20	19	19	20	49	28	17
T	18	18	21	19	20	20	21	20	20	18	22	22	20	16	22	18	19	18	16	18	16	19	17	17	17	18	20	22	16	19	14	18	18	20	15	15	12	6	11	6
G	30	30	32	31	26	31	29	27	31	28	31	32	29	31	29	32	35	32	30	31	26	32	36	28	28	31	36	31	32	26	28	36	29	26	42	28	22	38	20	26
20	36	34	29	33	35	31	32	32	29	34	25	30	35	37	30	32	27	32	36	33	35	30	31	33	46	31	29	34	35	32	38	24	32	34	25	38	45	7	40	51
tot	C	C	G	C	C	C	C	C	G	C	G	G	C	C	C	C	G	G	C	C	C	G	G	C	C	G	G	C	C	C	C	G	C	C	G	C	C	A	C	C

**Table 2:** Top Heart A 50% and Top Heart B 50%. Top Heart A 50% is made from the first 20 nucleotides from 5′UTR Top Heart and the last 20 nucleotides of 5′UTR Top CMs. Top Heart B is made from the last 20 nucleotides from 5′UTR Top Heart and the first 20 nucleotides of 5′UTR Top CMs.

Top 1000 Genes Sorted by Total Expression in Heart (n = 1000)																																								
Base/Pos	−40	−39	−38	−37	−36	−35	−34	−33	−32	−31	−30	−29	−28	−27	−26	−25	−24	−23	−22	−21	−20	−19	−18	−17	−16	−15	−14	−13	−12	−11	−10	−9	−8	−7	−6	−5	−4	−3	−2	−1
The best 5′UTR	C	C	C	C	C	C	C	C	C	C	G	C	G	C	C	C	G	G	C	G	C	C	C	C	C	G	C	C	C	C	C	G	C	C	G	C	C	A	C	C
The worse 5′UTR	C	C	C	C	C	C	C	G	G	C	G	G	C	C	G	C	G	G	C	G	G	G	G	C	C	C	G	C	C	C	C	G	C	C	G	C	C	A	C	C

**Table 3:** List of different 5′UTRs for the modRNA of nGFP or Luc that was used in this study.

1. 5′UTRcontrol: AAATAAGAGAGAAAATAAGAGTAAGAAGAAATATAAGAGCCACC
2. 5′UTR Top Heart: CCCCCCCCCCGCCCCCGGCGCCCCCGCCCCCGCCGCCACC
3. 5′UTR Top CMs: CCCCCCCGGCGGCCGCGGCGGGGCCCGCCCCGCCGCCACC
4. 5′UTR Top Elevated Heart: CCGCCCCCGCGGCCCCGGCCCGGCCGGCCCCGCCGCCACC
5. 5′UTR Top Heart A 50%: CCCCCCCCCCGCCCCCGGCGGGGCCCGCCCCGCCGCCACC
6. 5′UTR Top Heart B 50%: CCCCCCCGGCGGCCGCGGCGCCCCCGCCCCCGCCGCCACC

**Table 4:** List of open reading frames (ORFs) used in this work.

Luc ORF	atggccgatgctaagaacattaagaagggccctgctcccttctaccctctggaggatggcac cgctggcgagcagctgcacaaggccatgaagaggtatgccctggtgcctggcaccattgcct tcaccgatgcccacattgaggtggacatcacctatgccgagtacttcgagatgtctgtgcgc ctggccgaggccatgaagaggtacggcctgaacaccaaccaccgcatcgtggtgtgctctga gaactctctgcagttcttcatgccagtgctgggcgccctgttcatcggagtggccgtggccc ctgctaacgacatttacaacgagcgcgagctgctgaacagcatgggcatttctcagcctacc gtggtgttcgtgtctaagaagggcctgcagaagatcctgaacgtgcagaagaagctgcctat catccagaagatcatcatcatggactctaagaccgactaccagggcttccagagcatgtaca cattcgtgacatctcatctgcctcctggcttcaacgagtacgacttcgtgccagagtctttc gacagggacaaaaccattgccctgatcatgaacagctctgggtctaccggcctgcctaaggg cgtggccctgcctcatcgcaccgcctgtgtgcgcttctctcacgcccgcgaccctattttcg gcaaccagatcatccccgacaccgctattctgagcgtggtgccattccaccacggcttcggc atgttcaccaccctgggctacctgatttgcggctttcgggtggtgctgatgtaccgcttcga ggaggagctgttcctgcgcagcctgcaagactacaaaattcagtctgccctgctggtgccaa ccctgttcagcttcttcgctaagagcaccctgatcgacaagtacgacctgtctaacctgcac gagattgcctctggcggcgccccactgtctaaggaggtgggcgaagccgtggccaagcgctt tcatctgccaggcatccgccagggctacggcctgaccgagacaaccagcgccattctgatta ccccagagggcgacgacaagcctggcgccgtgggcaaggtggtgccattcttcgaggccaag gtggtggacctggacaccggcaagaccctgggagtgaaccagcgcggcgagctgtgtgtgcg cggccctatgattatgtccggctacgtgaataaccctgaggccacaaacgccctgatcgaca aggacggctggctgcactctggcgacattgcctactgggacgaggacgagcacttcttcatc gtggaccgcctgaagtctctgatcaagtacaagggctaccaggtggccccagccgagctgga gtctatcctgctgcagcaccctaacattttcgacgccggagtggccggcctgcccgacgacg atgccggcgagctgcctgccgccgtcgtcgtgctggaacacggcaagaccatgaccgagaag gagatcgtggactatgtggccagccaggtgacaaccgccaagaagctgcgcggcggagtggt gttcgtggacgaggtgcccaagggcctgaccggcaagctggacgcccgcaagatccgcgaga tcctgatcaaggctaagaaaggcggcaagatcgccgtgtaa
nGFP ORF	atggtgagcaagggcgaggagctgttcaccggggtggtgcccatcctggtcgagctggacgg cgacgtaaacggccacaagttcagcgtgtccggcgagggcgagggcgatgccacctacggca agctgaccctgaagttcatctgcaccaccggcaagctgcccgtgccctggcccaccctcgtg accaccctgacctacggcgtgcagtgcttcagccgctaccccgaccacatgaagcagcacga cttcttcaagtccgccatgcccgaaggctacgtccaggagcgcaccatcttcttcaaggacg acggcaactacaagacccgcgccgaggtgaagttcgagggcgacaccctggtgaaccgcatc gagctgaagggcatcgacttcaaggaggacggcaacatcctggggcacaagctggagtacaa ctacaacagccacaacgtctatatcatggccgacaagcagaagaacggcatcaaggtgaact tcaagatccgccacaacatcgaggacggcagcgtgcagctcgccgaccactaccagcagaac acccccatcggcgacggccccgtgctgctgcccgacaaccactacctgagcacccagtccgc cctgagcaaagaccccaacgagaagcgcgatcacatggtcctgctggagttcgtgaccgccg ccgggatcactctcggcatggacgagctgtacaagggagatccaaaaaagaagagaaaggta ggcgatccaaaaaagaagagaaaggtaggtgatccaaaaaagaagagaaaggtataa

## Data Availability

All modified mRNA (modRNA) vectors containing genes of interest noted in this paper will be made available to other investigators. My institution and I will adhere to the NIH Grants Policy on Sharing of Unique Research Resources including the “Sharing of Biomedical Research Resources: Principles and Guidelines for Recipients of NIH Grants and Contracts” issued in December 1999. Specifically, material transfers will be made with no more restrictive terms than in the Simple Letter Agreement or the UBMTA and without reach-through requirements. Should any intellectual property arise, which requires a patent, we would ensure that the technology remains widely available to the research community in accordance with the NIH Principles and Guidelines.

## References

[1] Mouilleron H., Delcourt V., Roucou X. (2016). Death of a dogma: Eukaryotic mRNAs can code for more than one protein. Nucleic Acids Res..

[2] van der Velden A.W., Thomas A.A. (1999). The role of the 5′ untranslated region of an mRNA in translation regulation during development. Int. J. Biochem. Cell Biol..

[3] Leppek K., Das R., Barna M. (2018). Functional 5′ UTR mRNA structures in eukaryotic translation regulation and how to find them. Nat. Rev. Mol. Cell Biol..

[4] Petibon C., Ghulam M.M., Catala M., Elela S.A. (2021). Regulation of ribosomal protein genes: An ordered anarchy. Wiley Interdiscip. Rev. RNA.

[5] Karikó K., Buckstein M., Ni H., Weissman D. (2005). Suppression of RNA recognition by Toll-like receptors: The impact of nucleoside modification and the evolutionary origin of RNA. Immunity.

[6] Karikó K., Muramatsu H., Welsh F.A., Ludwig J., Kato H., Akira S., Weissman D. (2008). Incorporation of pseudouridine into mRNA yields superior nonimmunogenic vector with increased translational capacity and biological stability. Mol. Ther..

[7] Pilishvili T., Gierke R., Fleming-Dutra K.E., Farrar J.L., Mohr N.M., Talan D.A., Krishnadasan A., Harland K.K., Smithline H.A., Hou P.C. (2021). Effectiveness of mRNA Covid-19 Vaccine among U.S. Health Care Personnel. N. Engl. J. Med..

[8] El Sahly H.M., Baden L.R., Essink B., Doblecki-Lewis S., Martin J.M., Anderson E.J., Campbell T.B., Clark J., Jackson L.A., Fichtenbaum C.J. (2021). Efficacy of the mRNA-1273 SARS-CoV-2 Vaccine at Completion of Blinded Phase. N. Engl. J. Med..

[9] Magadum A., Kurian A.A., Chepurko E., Sassi Y., Hajjar R.J., Zangi L. (2020). Specific Modified mRNA Translation System. Circulation.

[10] Labonia M., Senti M.E., van der Kraak P., Brans M., Dokter I., Streef T., Smits A., Deshantri A., de Jager S., Schiffelers R. (2024). Cardiac delivery of modified mRNA using lipid nanoparticles: Cellular targets and biodistribution after intramyocardial administration. J. Control. Release.

[11] Ascanelli C., Dahir R., Wilson C.H. (2024). Manipulating Myc for reparative regeneration. Front. Cell Dev. Biol..

[12] Wang A.Y.L., Chang Y.-C., Chen K.-H., Loh C.Y.Y. (2024). Potential Application of Modified mRNA in Cardiac Regeneration. Cell Transplant..

[13] Li S., Shen S., Xu H., Cai S., Yuan X., Wang C., Zhang X., Chen S., Chen J., Shi D.-L. (2023). IGF2BP3 promotes adult myocardial regeneration by stabilizing MMP3 mRNA through interaction with m6A modification. Cell Death Discov..

[14] Warren L., Manos P.D., Ahfeldt T., Loh Y.-H., Li H., Lau F., Ebina W., Mandal P.K., Smith Z.D., Meissner A. (2010). Highly efficient reprogramming to pluripotency and directed differentiation of human cells with synthetic modified mRNA. Cell Stem Cell.

[15] Di Cesare M., Perel P., Taylor S., Kabudula C., Bixby H., Gaziano T.A., McGhie D.V., Mwangi J., Pervan B., Narula J. (2024). The Heart of the World. Glob. Heart.

[16] Magadum A., Kaur K., Zangi L. (2019). mRNA-Based Protein Replacement Therapy for the Heart. Mol. Ther..

[17] Sultana N., Hadas Y., Sharkar M.T.K., Kaur K., Magadum A., Kurian A.A., Hossain N., Alburquerque B., Ahmed S., Chepurko E. (2020). Optimization of 5′ Untranslated Region of Modified mRNA for Use in Cardiac or Hepatic Ischemic Injury. Mol. Ther.—Methods Clin. Dev..

[18] Hinnebusch A.G., Ivanov I.P., Sonenberg N. (2016). Translational control by 5′-untranslated regions of eukaryotic mRNAs. Science.

[19] Ma Q., Zhang X., Yang J., Li H., Hao Y., Feng X. (2024). Optimization of the 5ʹ untranslated region of mRNA vaccines. Sci. Rep..

[20] Anttila V., Saraste A., Knuuti J., Hedman M., Jaakkola P., Laugwitz K.-L., Krane M., Jeppsson A., Sillanmäki S., Rosenmeier J. (2023). Direct intramyocardial injection of VEGF mRNA in patients undergoing coronary artery bypass grafting. Mol. Ther..

[21] Asrani K.H., Farelli J.D., Stahley M.R., Miller R.L., Cheng C.J., Subramanian R.R., Brown J.M. (2018). Optimization of mRNA untranslated regions for improved expression of therapeutic mRNA. RNA Biol..

[22] Castillo-Hair S., Fedak S., Wang B., Linder J., Havens K., Certo M., Seelig G. (2024). Optimizing 5′UTRs for mRNA-delivered gene editing using deep learning. Nat. Commun..

[23] Trepotec Z., Aneja M.K., Geiger J., Hasenpusch G., Plank C., Rudolph C. (2019). Maximizing the Translational Yield of mRNA Therapeutics by Minimizing 5′-UTRs. Tissue Eng. Part A.

[24] Haimov O., Sinvani H., Dikstein R. (2015). Cap-dependent, scanning-free translation initiation mechanisms. Biochim. Biophys. Acta (BBA)—Gene Regul. Mech..

[25] Chu Y., Yu D., Li Y., Huang K., Shen Y., Cong L., Zhang J., Wang M. (2024). A 5′ UTR Language Model for Decoding Untranslated Regions of mRNA and Function Predictions. Nat. Mach. Intell..

[26] Cao J., Novoa E.M., Zhang Z., Chen W.C.W., Liu D., Choi G.C.G., Wong A.S.L., Wehrspaun C., Kellis M., Lu T.K. (2021). High-throughput 5′ UTR engineering for enhanced protein production in non-viral gene therapies. Nat. Commun..

[27] Sample P.J., Wang B., Reid D.W., Presnyak V., McFadyen I.J., Morris D.R., Seelig G. (2019). Human 5′ UTR design and variant effect prediction from a massively parallel translation assay. Nat. Biotechnol..

[28] Wang K., Lee P., Mirams G.R., Sarathchandra P., Borg T.K., Gavaghan D.J., Kohl P., Bollensdorff C. (2015). Cardiac tissue slices: Preparation, handling, and successful optical mapping. Am. J. Physiol. Heart Circ. Physiol..

[29] Wiechert S., El-Armouche A., Rau T., Zimmermann W.-H., Eschenhagen T. (2003). 24-h Langendorff-perfused neonatal rat heart used to study the impact of adenoviral gene transfer. Am. J. Physiol. Circ. Physiol..

